# Chemical Characterization of the Oil Separated by Mechanical Pressing from *Strychnos madagascariensis* Dried Fruit Pulp Flour

**DOI:** 10.3390/foods11030474

**Published:** 2022-02-06

**Authors:** Sandra S. I. Chemane, Susana Casal, Rebeca Cruz, Teresa Pinho, Maida Khan, Olívia Pinho, Olga Viegas

**Affiliations:** 1 Faculdade de Ciências da Nutrição e Alimentação da Universidade do Porto, 4150-180 Porto, Portugal; s.chemane.se@gmail.com (S.S.I.C.); olivia.pinho@fcna.up.pt (O.P.); olgaviegas@fcna.up.pt (O.V.); 2 LAQV/REQUIMTE, Laboratório de Bromatologia e Hidrologia, Departamento de Ciências Químicas, Faculdade de Farmácia da Universidade do Porto, 4050-313 Porto, Portugal; rcruz@ff.up.pt (R.C.); teresapinho847@gmail.com (T.P.); 3 Departamento de Engenharia Rural, Faculdade de Agronomia e Engenharia Florestal, Universidade Eduardo Mondlane, Maputo 257, Mozambique; 4 Departamento de Engenharia Química, Faculdade de Engenharia, Universidade Eduardo Mondlane, Maputo 257, Mozambique; maida.khan@uem.ac.mz

**Keywords:** native fruits, *Strychnos madagascariensis*, high-oleic oil, monounsaturated oil, monkey fruit, sterols

## Abstract

In Mozambique, rural communities produce flours from the dried pulp of *Strychnos madagascariensis* fruits. Owing to its high lipid content, the oil from this flour is frequently separated by pressing to be used as seasoning and medicine. Aiming to characterize this oil, flour samples (*n* = 24), dried at two different temperatures (55 °C and 65 °C), were collected from four local communities, together with a control sample prepared in the lab (50 °C). The resulting oil was fluid at room temperature, deep orange, and characterized by a high content of oleic acid (62–63%), followed by palmitic (20%) and linoleic (7%). It contained considerable amounts of tocols (25–34 mg/100 g) and carotenoids (8–10 mg/100 g), as well as sterols (431 ± 10 mg/100 g) and triterpenic alcohols (823 ± 4 mg/100 g mg/100 g). The overall composition was highly consistent between origins and temperatures, with only small statistically significant differences (*p* < 0.05), mostly between the community dried flours and control group. However, its high free fatty acid content (22–25%) reveals intensive enzymatic hydrolysis during the drying/fermentation steps, whose extension can be reduced by optimizing its technological process. Its chemical profile supports some of its folklore uses, revealing that it can be a promising source of edible oil, with health and technological potential that is worth optimizing and exploring.

## 1. Introduction

The *Strychnos* genus belongs to the Loganiaceae family, indigenous to tropical and subtropical Africa. The most consumed species, which are prevalent in woodlands of Southern Africa, are *S. innocua*, *S. madagascariensis*, *S. cocculoides*, *S. pungens,* and *S. spinosa*. *S. madagascariensis* fruits, known as monkey orange, are usually eaten raw, or fermented and dried by sun exposure and used to make alcoholic beverages, and the pulp produces an appreciated sweet when mixed with honey or in the form of tea [1,2,3]. Due to their abundance, the preservation of *Strychnos* sp. fruits is recognized as an important strategy to enhance food availability in times of shortage, reducing losses and underutilization [4]. Local populations also report its use as a multipurpose folk medicine [5], including its folklore use in the management of diabetes mellitus and hypertension [6]. In the southern part of Mozambique, where the fruit is called “macuácua” (“Makwakwa”), it is usually eaten after being transformed into a flour-like product obtained from the dried pulp (“nfuma”), especially in times of scarcity of basic foods. Some communities extract a liquid oil with intense orange-brown color from “nfuma” with several applications, including cooking purposes.

Edible vegetable oils are essential in people’s daily diet and can be produced from seeds, nuts, kernels, beans, cereals, and fruits pulp (mesocarp). The most widely consumed oil worldwide is palm oil, also obtained from a fruit pulp, followed by other important sources such as soybean, corn, sunflower, peanut, rapeseed, and olives, among others [7]. Oils extracted from plant sources have a successful history of use by rural communities as a food ingredient, as well as in medicine, cosmetic, and fuel applications. The continuing seek for innovation in ingredient sources and the demand for natural alternatives increases the opportunity for indigenous African vegetable oils. Some oils pressed from seeds of African trees have become popular ingredients in cosmetic formulations [8], or for food purposes, as the oil from seeds of *Moringa oleifera* [9] or avocado pulp [10], relatively new in culinary circles. As far as the authors are aware, no detailed characterization of the oil extracted from the “nfuma” flour has been reported so far.

This research aims to determine the compositional basis of the oils physically extracted from the dry pulp of *S. madagascariensis* fruits produced following traditional procedures while evaluating its nutritional and technological potential. Gathering local communities’ knowledge is important and useful to determine the true value of their practices and will lead to more rational decisions about its sustainable utilization and valorization.

## 2. Materials and Methods

### 2.1. Reagents and Standards

The reagents used in this work were of analytical or chromatographic grade, obtained from several suppliers. A certified fatty acids methyl ester (FAME) standard mixture (Supelco 37 Component FAME Mix, CRM 47885, TraceCERT®, Supelco, Bellefonte, PA, USA) was used. All sterols’ standards (cholesterol, campesterol, stigmasterol, ß-sitosterol, and stigmasterol) were purchased from Sigma–Aldrich as were the mono-, di-, and triacylglycerols reference standards, β-carotene, lutein, retinol, and retinol acetate. Tocopherols and tocotrienols standards (α, β, λ, and δ) plus α-tocopherol palmitate and acetate were acquired from Supelco (Bellefonte, PA, USA) and Larodan (Solna, Sweden), and the internal standard tocol (2-Methyl-2-(4,8,12-trimethyltridecyl)-chroman-6-ol) was purchased from (Matreya, Inc., State College, PA, USA). The HPLC solvents n-hexane, dioxane, and tetrahydrofuran (THF) were from Merck (Darmstadt, Germany).

### 2.2. Sampling and Oil Extraction

The oils were extracted mechanically from flours produced in four districts in southern Mozambique Marracuene, Manhiça, Chókwé, and Chicualacuala. The flour was obtained from the pulp of ripe fruits collected in the summer season, sun-dried for 2 to 4 days to ease seed separation, and then slightly toasted in a metallic tray at two temperature ranges (50–60 °C and 60–70 °C) for one hour. The process was performed in triplicate on different days, for both temperatures, on each community, resulting in six independent samples of 5 kg per community, in a total of 24 independent community samples. The seeds were separated from the dried pulp, which was further crushed manually using a mortar and pestle until the flour was obtained. All processed flours were stored at room temperature in closed containers for later extraction of the oil in a central laboratory. Additionally, three fresh fruit batches collected in Marracuene were processed in the laboratory, being dried at 50 °C for two days, and were used as control for process variability. All the samples were verified for moisture content by oven drying at 100 °C, ranging from 3.6% to 5.2%.

For oil extraction purposes, the flour (500 g portions) was warmed using an oven (Memmert UN 110) at 40 °C for 30 min, and oil extraction was performed using a hydraulic hand press machine (Carver Menomonee Falls, WIS 53051 USA Serie 24000–103). The oils were filtered with Whatman 40 paper and anhydrous sodium sulphate to remove all moisture and solid impurities, placed in glass containers, closed, and protected with aluminum foil and stored at 4 ± 1 °C until analysis. Oil extraction yield after mechanical pressing was not evaluated, but the initial fat content of the flours was evaluated by Soxhlet extraction with petroleum ether (40–60 °C), ranging from 25.7 to 32.3%.

### 2.3. Fatty Acids Composition

Esterified fatty acids were evaluated as methyl esters, after alkaline trans-esterification using potassium hydroxide (2M solution in methanol) [11] on a Chrompack CP9001 system (Middelburg, The Netherlands), with flame ionization detection (FID). Fatty acid separation was carried out on a Select FAME (50 m × 0.25 mm × 0.25 μm) column (Agilent, Santa Clara, CA, USA), with helium as carrier gas (1 mL/min) and a temperature ranging from 120 to 220 °C. The injector (split 1:50) and detector were set at 250 and 270 °C, respectively. The results were expressed in relative percentage of each fatty acid methyl ester in the total fatty acids, after standardization of the detector response with a certified reference standard.

### 2.4. Triacylglycerols, Diacylglycerols, and Free Fatty Acids

Total triacylglycerols (TG), diacylglycerols (DG), and free fatty acids (FFA) contents were analyzed by size-exclusion high-performance liquid chromatography (HPSEC) following ISO 18395:2005 [12] on a HPLC system from Jasco (Japan) equipped with a refractive index detector (132 RI Detector, Gilson, Saint-Avé, France). Separation was achieved on a Phenogel 5 µm 100Å column (600 × 7.8 mm; Phenomenex, Torrance, CA, USA), with THF at a flow rate of 1 mL/min. An accurate oil mass was diluted in THF and homogenized by stirring before injection. The results were expressed in relative percentage, after calibration of the detector responses with adequate standards.

### 2.5. Phytosterols

The sterol and triterpenic alcohol fraction was separated by thin-layer chromatography (TLC) after saponification, followed by silylation [11]. Quantification was obtained by gas chromatography with FID detection (TRACE GC; Thermo Finnigan, Rodano, Italy) after separation in an DB-5MS column (30 m × 0.250 mm, 0.25 µm; Agilent J&W, USA), with a temperature gradient from 250 to 280 °C and helium at a flow rate of 1.0 mL/min. Injection (split ratio 1:10; 280 °C) was performed with an automatic injector (Thermo Scientific Al 1310, Milan, Italy). Results for individual sterols were reported in relative percentage, while total sterols were reported in mg/100 g, using betulin as an internal standard, as were the triterpenic alcohols. Identification of the compounds was confirmed by GC-MS, using an Agilent GC 6890N chromatograph equipped with a mass-selective detector (5977B MSD), with electron ionization of 70 eV set in the full scan mode (m/z 50−650) with equivalent chromatographic condition from those indicated above. Temperatures of interphase, ionization source, and quadrupole were 300, 230, and 150 °C, respectively. The compounds were identified by spectra comparison with authentic standards, using the NIST 11 mass spectra library and literature data. Only the low-temperature processed samples were evaluated for phytosterols.

### 2.6. Tocols and Carotenes

Tocols and carotenes were analyzed by HPLC with fluorescence and diode-array detection, respectively (Jasco, Tokyo, Japan) [13]. An accurate oil solution was prepared in n-hexane, after addition of the internal standard solution and homogenization by stirring. For separation, a normal phase silica column was used (Supelcosil TM LC-SI; 7.5 cm × 3 mm; 3 µm) (Supelco, Bellefonte, PA, USA), conditioned at 25 °C and eluted with a gradient of 1,4-dioxane and *n*-hexane at a flow rate of 0.75 mL/min. Detection was programmed for excitation at 290 nm and emission at 330 nm for tocols and at 446 nm for carotenes. Tocols were tentatively identified using commercial standards and their UV spectra. The main unidentified tocol-like compound was quantified with a calibration curve of β-tocotrienol, the closest tocol in terms of retention time. Carotenoids were tentatively identified by their typical UV spectra, based on their absorbance maxima, III/II ratios, and spectra shoulders, as well as by comparing to available commercial standards. All carotenoid-like compounds were grouped and quantified in mg of β-carotene equivalents/100 g of oil. GC-MS was further used for the tentative confirmation of the tocol-like compound detected in the oil samples, after silylation with N,O-bis(trimethylsilyl)trifluoroacetamide (BSTFA), using an HP5-MS column (30 m × 0.25 mm I.D. × 0.25 μm film thickness, Agilent J&W), on the same GC-MS equipment described above, using all the previously mentioned standards and the available spectra from the NIST library.

### 2.7. Statistical Analysis

The statistical outcomes resulting from the chemical analysis are shown as average values and standard deviation of each community/temperature sample group, with duplicate chemical analysis of each sample (*n* = 3 × 2). To compare the lipid profile of the flour oil from the different communities, normal distribution of the residuals was determined by the Shapiro−Wilk test (sample size < 50) and the homogeneity of variances through Levene’s test. Afterwards, dependent variables were studied using a one-way and two-way analysis of variance (ANOVA), subjected or not to Welch correction, followed by Duncan’s or Dunnett’s T3 test, depending on whether the requirement of the homogeneity of variances was verified or not, respectively.

Principal component analysis (PCA) was applied to emphasize variation and bring out strong patterns in a data set according to the different lipid main constituents. For this purpose, data were previously standardized (z-scores) and oblimin with Kaiser normalization was selected as the rotation method considering that dependent variables were highly correlated. Statistical analyses were performed at a 5% significance level using SPSS software (version 27.0, IBM Corporation, New York, NY, USA).

## 3. Results and Discussion

The oils obtained from the *S. madagascariensis* fruit flours prepared from the four communities (Marracuene, Manhiça, Chókwé and Chicualacuala) were characterized for fatty acids composition, glycerides, phytosterols, tocols and carotenes.

### 3.1. Fatty Acids Composition

The fatty acid composition, detailed in Table 1, shows a high-oleic oil, with a very consistent content of oleic acid, ranging from 62.4 to 62.7%, followed by the saturated palmitic (19.4 to 20.1%) and stearic acids (4.3 to 4.6%). The only polyunsaturated fatty acids detected were linoleic acid (6.8–7.1%) and α-linolenic (1.7–1.8%). The amount of short-chain saturated fatty acids was low (<0.3%), as is the sum of long-chain ones (arachidic, behenic, and lignoceric, <1.2%).

The fatty acid profile, with oleic acid as major fatty acid, is similar to that of other natural high-oleic crude oils, such as almond, hazelnut, pistachio, avocado and olive oil, as this fatty acid largely is responsible for the unique nutritional impact of these oils, their great stability to oxidation [10,14], and contributing to the liquid appearance of these oil at room temperature. High-oleic oils have also been shown to have beneficial impacts on serum cholesterol and low-density lipoproteins without affecting the high-density ones, thus minimizing the risk of cardiovascular disease, already with approved health claims on cardiovascular health [15]. On the other hand, the amount of saturated fatty acids (25.9–26.6%) is higher than the amount present in the above-mentioned high-oleic oils but well below typical tropical fats such as palm or coconut.

In comparison with other oils of African origin, macuácua oil has a higher content of oleic acid than *Adansonia digitata* (baobab) (30 to 42%), *Citrullis lantus* (13 to 17%), *Schinziophyton rautanenii* (15 to 19%), or *Trichilia emética* (15%) but is similar to *Simenia africana* (54 to 72%) and *Sclerocarya birrea* seed oil (70 to 78%). These oils are used in dermatological issues, among others [16,17,18].

In terms of variability between communities, the results indicate a high consistency between all the communities studied. When the two temperature ranges (50–60 °C and 60–70 °C) used in the communities are compared, only minor differences are observed within each community and only in the most sensible fatty acids, namely in linolenic acid, with significantly (*p* < 0.05) lower amounts in the samples processed at higher temperatures in both Marracuene and Chicualacuala, but not in the other two communities. The *trans* fatty acid content, known to increase with temperature, was significantly higher in the Chókwé samples processed at higher temperatures against those processed at lower temperatures, but no significant differences were observed in the other communities. However, significant differences between communities and the control samples were verified (*p* < 0.05): the control group revealed a higher content of both linoleic and linolenic acid and long-chain SFA, while showing lower contents of palmitic and stearic as well as reduced content of total *trans* fatty acids. Altogether, this highlights a lower fatty acid oxidation and a less aggressive procedure, as expected from the more controlled drying process performed in the lab, as well as lower temperature.

### 3.2. Triacylglycerols, Diacylglycerols, and Free Fatty Acids

The results in Table 2 illustrate that triacylglycerols are the most important glyceride forms in the oil, ranging from 65 to 75%, while the diglycerides vary from 8 to 10%. However, the oil has an extremely high content of free fatty acids, ranging from 17 to 26%. Although FFA are typical in crude, non-refined oils [19], these amounts reveal an intense lipolytic activity during the preparation of the flour, or already in the mature fruit, with glyceride hydrolysis, most likely induced by natural lipases in the pulp, although some microbial activity cannot be excluded. Lipolysis is also a concern in palm oil, with a rapid lipolytic activity after picking, particularly in ripe fruits. In palm oil, the free fatty acid content is a determinant for its quality and economic revenue, since these free fatty acids should be removed by refining, with a limit of edibility of only 5% in this oil, well below the amounts determined for the oil under study, therefore questioning its edibility. To reduce this enzymatic activity in palm oil processing, a rapid enzyme inactivation by sterilization is performed after fruit picking. A similar approach could be tested with these fruits, but a recommendation to pick the fruits before being fully ripe can also potentially contribute to reduce hydrolysis [20]. Both procedures could contribute to enhance the quality and edibility of this oil and deserve further testing.

Overall, there are no significant differences (*p* > 0.05) in the results of all communities’ samples, even with different drying temperatures, except in Manhiça for the samples processed at the higher temperatures. Interestingly, these samples presented significantly lower amounts of FFA, and consequently higher triacylglycerols, with percentages close to the ones obtained in the laboratory samples. Therefore, the temperature was not the determinant factor in this hydrolysis extent, because lab samples were the ones processed at the lower temperatures. The sun-drying step extension is influenced by weather conditions and thus is likely the main determinant of this hydrolysis extension, with probable enzymatic inactivation at the roasting step, for all the temperatures tested. The time taken to inactivate the enzymes is probably the main determinant for glyceride hydrolysis. Since the time between fruit picking and its processing in the lab was also not under full control, hydrolysis might still have occurred in the control samples. Nevertheless, the high consistency in the hydrolysis values between all the samples is indicative that time/temperature parameters will have minor effects on hydrolysis extension and that more drastic measures should be implemented.

### 3.3. Minor Bioactive Constituents

Carotenoids are responsible for the oil’s typical orange color but are also known to protect the lipids from oxidation because of their ability to act as effective quenchers of singlet oxygen [21]. The vitamin A profile in the oil was dominated by carotenes, with minor amounts of non-identified carotenoid-like compounds, all quantified as β-carotene equivalents (Table 2). The analytical method used does not distinguish α and β-carotenes, so the carotenes are expressed on a β-carotene basis but might correspond to a sum of different carotenes. The total amounts of this group ranged from 8 to 13 mg/100 g (Table 2). These carotene amounts are superior to other crude vegetable oils [22], supporting its orange color, despite being lower than typical palm oil amounts (50–70 mg/100 g) [7], recognized as one of the highest food sources for carotenes.

Apparent variability was observed between communities but of no statistical significance. As for differences imposed by the processing temperatures, again no differences were observed, except in Manhiça, with significantly higher carotene amounts in the samples processed at the higher temperatures, even higher than the control one. This is indicative of higher carotene protection, being consistent with the lower lipolysis already discussed above for this sample group.

Vitamin E includes tocopherols and tocotrienols. Although tocopherols are most frequently found in vegetable oils, with α-tocopherol being generally the most abundant in foods, tocotrienols are also typical in some vegetables. Macuácua oil is characterized by an atypical profile because only vestigial amounts of α-tocopherol and tocopherol esters were detected (not quantified), and the chromatograms were dominated by a single main compound. This unknown compound elutes in our normal-phase chromatographic system between β- and γ-tocotrienols has a maximum absorbance at 296 nm and the typical fluorescence of these tocols, but no true identification was possible, even by GC-MS analysis after silylation and cross checking with analytical standards nor the NIST library. Knowing that palm oil is also rich in tocotrienols, and due to its chromatographic behavior, we have quantified it in β-tocotrienol equivalents with total amounts between 24 and 35 mg/100 g (Table 2). Several studies have demonstrated that tocotrienols have highly interesting activities in health and disease that are distinct from those of tocopherols [23], representing another interesting potential health feature for this oil. Even without clarification of the compound’s identity, the amounts are within the ranges reported for crude vegetable oils [14]. The isolation and identification of this compound is worth exploring in future works.

When the communities are compared, the amounts were highly consistent, except again for Manhiça samples processes at the higher temperatures, with significantly higher amounts, equivalent to those prepared in the laboratory. Again, higher temperatures were not responsible for tocol losses.

The composition of the phytosterols in the *S. madagascariensis* oil was highly consistent between all the samples processed at the lower temperatures for each community, containing sterols and triterpene alcohols. Total sterols were present with 431 ± 10 mg/100 g, characterized by relative percentages of 30.5 ± 0.4% for β-sitosterol, 30.4 ± 0.2% of campesterol, 21.4 ± 0.2% of ∆5-avenasterol, and 17.7 ± 0.7% of stigmasterol. Two triterpene alcohols (4,4-dimethylsterols) were present in high amounts, identified by GC-MS after conversion to trimethylsilyl ethers as cycloartenol and 24-methylenecycloartanol on the basis of their mass fragmentation pattern (m/z), retention index, and match (>950) and literature [24]. These compounds summed up to 823 ± 9 mg/100 g on average, with a very consistent content between all the samples and proportion between them of 2.4–2.7 to 1. The available results are detailed in Table 2. The global amounts of sterols can be considered high within the most common vegetable oil, being higher than soybean, sunflower, peanut, or olive oils, and similar to sesame and flaxseed oils, but still lower then corn, rapeseed, or rice bran oils. Their relative profile is distinct from the most common vegetable oils, with flaxseed as the most similar one [14,25]. As for the triterpenic alcohols, their amounts can be regarded as very high in comparison with the most common vegetable oils, being higher than rice bran oil or *Camellia oleifera* oil, which is typical in Asia and is recognized as one of the highest sources [24]. Vestigial amounts of β-amyrin and squalene were also detected but not quantified.

The presence of these compounds is interesting from a health perspective. While phytosterols in general are considered relevant to reducing atherogenesis-related risks, these triterpene alcohols have been identified as having antidiabetic properties [26] among the health effects reported for this fruit.

### 3.4. Samples Classification

**Figure 1:**
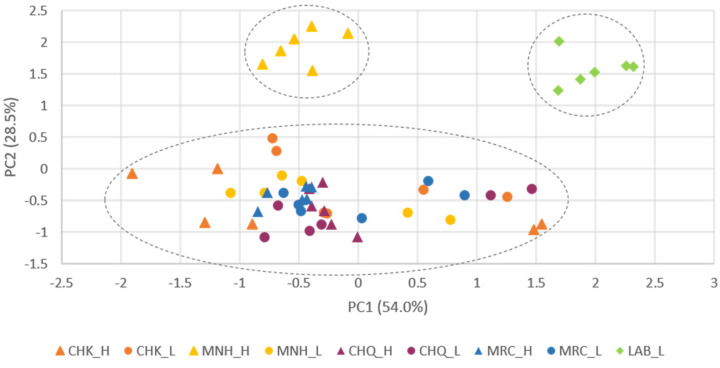
Principal component analysis of flour oils produced at Marracuene (MRC), Manhiça (MNH), Chókwé (CHK), Chicualacuala (CHQ), and lab (control) at low (L 50–60 °C) and high (H 60–70 °C) temperatures.

With the data acquired from oil samples, two different statistical tools were applied in order to tentatively investigate the effect of communities and roasting temperatures on the lipid composition by a multivariate two-way ANOVA test (Table 3) and to classify the oils based on their chemical profile by a PCA test (Figure 1).

First, the analysis of variance outcomes revealed, in general, significant effects of community and temperature, as well as a significant interaction between both factors for glycerides, FFA, carotenes, and tocols in oils samples (Table 3). However, this was not observed for the main classes of fatty acids, except that total *trans* were significantly influenced by communities and interaction, while total MUFAs showed a moderate influence from temperature and the interaction. Communities seemed to play a major role in the content of total *trans* (Z = 17.876, *p* < 0.001), carotenes (Z = 16.572, *p* < 0.001), and tocols (Z = 31.541, *p* < 0.001), but extraction temperature was a key factor for the prevalence of triglycerides (Z = 22.679, *p* < 0.001) and FFA (Z = 20.134, *p* < 0.001). Moreover, the interaction between these two variables show greater effect on FFA (Z = 10.606, *p* < 0.001), carotenes (Z = 24.846, *p* < 0.001), and tocols (Z = 15.266, *p* < 0.001) in comparison with the remaining micronutrients studied herein.

Hence, for the majority of the analyzed substances, both communities and the temperature used for the extraction are determinants for the variability of the composition of the oils.

A PCA test was conducted to classify the communities and production processes based on their lipid composition. The outcomes of dimension reduction analysis explained 82.5% of the total data variance by proving two principal components, as represented in Figure 1, whose variable communalities were all higher than 0.709. Three clusters were identified: one corresponding to the control sample, another to the Manhiça community (high temperature), and a third one to the remaining samples. The first principal component (PC1) factor, which comprised 54.0% of the total variance, can separate two groups: all the community samples located in the neutral and negative region, with high dispersion, and the control positioned in the positive region, with reduced overlapping. This finding may be correlated with the fact that the control samples exhibited the highest values for linoleic acid (PC1 loading = 0.946) and linolenic acid (PC1 loading = 0.949) and a lower content of SFA (PC1 loading = −0.915). The second principal component (PC2) factor, which accounted for 28.5% of the total variance observed, was also able to separate the control and Manhiça (high temperature) from the remaining samples, probably due to the higher content of tocols (PC2 loading = 0.894) and carotenes (PC2 loading = 0.890) and a lower content of FFA (PC2 loading = −0.828).

## 4. Conclusions

This research aimed to determine the composition and quality of *S. madagascariensis* pulp oil, obtained in local communities in Mozambique using a traditional process involving the dried and grounded pulp flour “nfuma”. The analysis revealed a high level of oleic oil, with high amounts of carotenoids, tocols, and phytosterols, which are important from a health and technological perspective. In addition, this study provides scientific evidence that the method of drying and extraction performed by the communities does not seem to impose variations in its composition, which is also close to the one obtained in the samples prepared in the laboratory. Its high content of non-esterified fatty acids, probably derived from lipases activity during drying, conditions its edibility and deserves to be explored, by either thermal inactivation or shorter drying period.

Fats are an integral part of a balanced and complete diet. Macuácua oil has a long tradition of consumption by local communities. Its nutritional attributes, namely its monounsaturated profile and richness in antioxidants and health-promoting substances, make it a fat that is worth exploring. However, its high lipolysis properties make it unsuitable for consumption. Technological solutions to improve the oil quality and shelf life and reduce lipolysis of this not yet fully exploited resource must be developed and could be exploited by agro-industry and become a source of income for poor rural areas in the future.

## Figures and Tables

**Table 1:** Fatty acid composition relative percentage (%) (mean ± standard deviation, *n* = 3 × 2) of oils extracted from the flour obtained at different temperatures from *Strychnos madagascariensis* fruits harvested and prepared by the communities of Chóckwé, Manhiça, Chicualacuala, and Marracuene, in southern Mozambique.

	Chókwé		Manhiça		Chicualacuala		Marracuene		Control
	50–60 °C	60–70 °C	50–60 °C	60–70 °C	50–60 °C	60–70 °C	50–60 °C	60–70 °C	50 °C *
C6:0	0.22 ± 0.09	0.28 ± 0.02	0.25 ± 0.02	0.25 ± 0.02	0.23 ± 0.02	0.21 ± 0.04	0.24 ± 0.03	0.25 ± 0.02	0.23 ± 0.01
C8:0	0.13 ± 0.02	0.13 ± 0.01	0.13 ± 0.01	0.12 ± 0.01	0.12 ± 0.01	0.11 ± 0.01	0.12 ± 0.01	0.13 ± 0.01	0.11 ± 0.01
C14:0	0.13 ± 0.01	0.15 ± 0.02	0.13 ± 0.01	0.13 ± 0.00	0.13 ± 0.00	0.13 ± 0.01	0.13 ± 0.01	0.13 ± 0.00	0.15 ± 0.01
C15:0	0.08 ± 0.01	0.09 ± 0.01	0.08 ± 0.00	0.08 ± 0.00	0.08 ± 0.01	0.08 ± 0.00	0.08 ± 0.01	0.08 ± 0.00	0.09 ± 0.00
C16:0	19.9 ± 0.24	20.1 ± 0.34	19.9 ± 0.22	20.0 ± 0.17	19.9 ± 0.27	19.9 ± 0.10	19.9 ± 0.19	20.0 ± 0.04	19.5 ± 0.08
C17:0	0.09 ± 0.00	0.10 ± 0.01	0.10 ± 0.01	0.10 ± 0.01	0.10 ± 0.01	0.10 ± 0.01	0.10 ± 0.01	0.10 ± 0.01	0.09 ± 0.01
C18:0	4.46 ± 0.09	4.45 ± 0.18	4.53 ± 0.09	4.57 ± 0.05	4.51 ± 0.07	4.61 ± 0.06	4.56 ± 0.11	4.54 ± 0.02	4.33 ± 0.04
C20:0	0.61 ± 0.01	0.64 ± 0.03	0.62 ± 0.01	0.62 ± 0.01	0.63 ± 0.02	0.63 ± 0.01	0.61 ± 0.01	0.62 ± 0.02	0.65 ± 0.01
C22:0	0.31 ± 0.01	0.30 ± 0.03	0.30 ± 0.01	0.30 ± 0.02	0.31 ± 0.01	0.32 ± 0.03	0.31 ± 0.02	0.32 ± 0.02	0.35 ± 0.01
C24:0	0.18 ± 0.01	0.18 ± 0.02	0.18 ± 0.02	0.18 ± 0.01	0.19 ± 0.02	0.19 ± 0.01	0.18 ± 0.01	0.19 ± 0.01	0.21 ± 0.01
**Total SFA**	**26.2 ± 0.3 ^a^**	**26.6 ± 0.5 ^A^**	**26.4 ± 0.3 ^a^**	**26.5 ± 0.2 ^A^**	**26.4 ± 0.3 ^a^**	**26.5 ± 0.1 ^A^**	**26.4 ± 0.2 ^a^**	**26.5 ± 0.1 ^A^**	**25.9 ± 0.1 ^bB^**
C16:1	1.59 ± 0.02	1.60 ± 0.04	1.58 ± 0.03	1.61 ± 0.02	1.59 ± 0.04	1.60 ± 0.02	1.60 ± 0.03	1.61 ± 0.01	1.55 ± 0.02
C18:1	62.74 ± 0.17	62.36 ± 0.17	62.67 ± 0.18	62.58 ± 0.14	62.53 ± 0.13	62.50 ± 0.09	62.61 ± 0.26	62.49 ± 0.06	62.66 ± 0.11
C20:1	0.30 ± 0.02	0.30 ± 0.03	0.31 ± 0.02	0.30 ± 0.02	0.31 ± 0.03	0.30 ± 0.01	0.30 ± 0.01	0.30 ± 0.01	0.34 ± 0.02
**Total MUFA**	**64.9 ± 0.1**	**64.5 ± 0.2 ^B^**	**64.7 ± 0.2**	**64.7 ± 0.1 ^A^**	**64.7 ± 0.1**	**64.6 ± 0.1 ^A^**	**64.7 ± 0.2**	**64.6 ± 0.1 ^A^**	**64.8 ± 0.1 ^A^**
C18:2	6.80 ± 0.08	6.85 ± 0.24	6.79 ± 0.07	6.76 ± 0.03	6.84 ± 0.13	6.78 ± 0.03	6.81 ± 0.10	6.75 ± 0.02	7.14 ± 0.06
C18:3	1.71 ± 0.04	1.71 ± 0.07	1.70 ± 0.04	1.70 ± 0.01	1.73 ± 0.06	1.72 ± 0.02	1.74 ± 0.05	1.70 ± 0.03	1.83 ± 0.02
**Total PUFA**	**8.7 ± 0.1 ^b^**	**8.7 ± 0.3 ^B^**	**8.7 ± 0.1 ^b^**	**8.7 ± 0.1 ^B^**	**8.8 ± 0.2 ^b^**	**8.7 ± 0.1 ^B^**	**8.8 ± 0.2 ^b^**	**8.7 ± 0.1 ^B^**	**9.2 ± 0.1 ^aA^**
**Total *trans***	**0.24 ± 0.01 ^a^**	**0.28 ± 0.02 ^A^**	**0.22 ± 0.02 ^b^**	**0.23 ± 0.02 ^B^**	**0.20 ± 0.03 ^b^**	**0.21 ± 0.02 ^C^**	**0.21 ± 0.03 ^b^**	**0.21 ± 0.01 ^BC^**	**0.18 ± 0.01 ^cC^**

* Prepared at the laboratory; different uppercase and lowercase letters in a row correspond to statistically significant (*p* < 0.05) differences between dependent variables regarding the effect of high and low extraction temperatures, respectively, for fatty acids main classes and individual PUFAs.

**Table 2:** Glycerides profile (% TG, DG, FFA), carotenes, tocols, sterols, and triterpene alcohols (mg/100 g) of the oil extracted from the flours obtained at different temperatures from *Strychnos madagascariensis* fruits (mean ± standard deviation, *n* = 3 × 2).

	Chókwé		Manhiça		Chicualacuala		Marracuene		Control
	50–60 °C	60–70 °C	50–60 °C	60–70 °C	50–60 °C	60–70 °C	50–60 °C	60–70 °C	50 °C *
Trialylglycerols (%)	66 ± 1 ^ab^	69 ± 5 ^B^	66 ± 1 ^ab^	75 ± 1^A^	67 ± 3 ^b^	67 ± 3 ^B^	65 ± 0 ^b^	66 ± 1 ^B^	74 ± 0 ^aA^
Diacylglycerols (%)	9 ± 1 ^b^	9 ± 1	9 ± 0 ^ab^	8 ± 0	9 ± 0 ^ab^	9 ± 0	10 ± 1 ^b^	9 ± 1	9 ± 1 ^b^
Free fatty acids (%)	25 ± 1 ^a^	22 ± 5 ^A^	25 ± 1 ^a^	17 ± 1 ^B^	24 ± 2 ^a^	24 ± 3 ^A^	26 ± 1 ^a^	25 ± 0 ^A^	17 ± 1 ^bB^
Carotenes ^#^ (mg/100 g)	9 ± 2 ^b^	8 ± 1 ^C^	10 ± 1 ^c^	14 ± 1 ^A^	8 ± 1 ^c^	8 ± 1 ^BC^	10 ± 1 ^c^	10 ± 1 ^B^	12 ± 1 ^aA^
Tocols ^##^ (mg/100 g)	30 ± 3 ^bc^	24 ± 2 ^D^	27 ± 2 ^b^	34 ± 0 ^A^	26 ± 1 ^c^	26 ± 2 ^D^	26 ± 3 ^b^	26 ± 1 ^C^	35 ± 2 ^aB^
Sterols ^###^ (mg/100 g)	435 ± 3 ^a^	-	423 ± 4 ^b^	-	427 ± 3 ^a^	-	427 ± 7 ^ab^	-	452 ± 4 ^c^
Triterpene alcohols ^####^ (mg/100 g)	826 ± 1	-	825 ± 9	-	818 ± 6	-	815 ± 12	-	826 ± 6

* Prepared at the laboratory. Sum of carotenes and non-identified carotenoid-like compounds, quantified in ^#^ β-carotene equivalents. ^##^ Unknown tocol-like compounds quantified in β-tocotrienol equivalents. ^###^ Sum of β-sitosterol, campesterol, ∆5-avenasterol and stigmasterol, quantified in betulin equivalents.^####^ Sum of cycloartenol and 24-methylenecycloartanol quantified in betulin equivalents. For sterols and triterpene alcohols, data available only for the low temperature samples. Different uppercase and lowercase letters in a row correspond to statistically significant (*p* < 0.05) differences between dependent variables regarding the effect of high and low extraction temperatures, respectively.

**Table 3:** Multivariate analysis of variance applied to lipid main classes.

	Communities			Extraction Temperatures			Interaction		
	Z	*p*	η^2^	Z	*p*	η^2^	Z	*p*	η^2^
Total SFA	0.023	0.995	0.002	3.102	0.085	0.064	1.843	0.153	0.109
Total MUFA	0.490	0.691	0.032	5.986	0.018	0.117	4.804	0.005	0.243
Total PUFA	0.954	0.423	0.060	0.035	0.852	0.001	0.530	0.664	0.034
Total *trans*	17.876	0.000	0.544	1.896	0.175	0.04	3.871	0.015	0.205
Triacylglycerols	8.395	0.000	0.359	22.679	0.000	0.335	9.766	0.000	0.394
Diacylglycerols	3.419	0.025	0.186	8.007	0.007	0.151	2.007	0.126	0.118
Free fatty acids	8.116	0.000	0.351	20.134	0.000	0.309	10.606	0.000	0.414
Carotenes	16.572	0.000	0.525	1.236	0.272	0.027	24.846	0.000	0.624
Tocols	31.541	0.000	0.678	8.221	0.006	0.154	15.266	0.000	0.504

## Data Availability

Data are contained within the article.
